# Polydatin Attenuates 14.1 MeV Neutron-Induced Injuries via Regulating the Apoptosis and Antioxidative Pathways and Improving the Hematopoiesis of Mice

**DOI:** 10.1155/2020/8905860

**Published:** 2020-08-31

**Authors:** Jiaming Guo, Tingting Liu, Long Ma, Wei Hao, Hongli Yan, Taosheng Li, Yanyong Yang, Jianming Cai, Fu Gao, Zhao Xu, Hu Liu

**Affiliations:** ^1^Department of Radiation Medicine, College of Naval Medicine, Naval Medical University, Shanghai 200433, China; ^2^Department of Reproductive Medicine Center, Changhai Hospital, Naval Medical University, Shanghai 200433, China; ^3^Department of Endocrinology, Changhai Hospital, Naval Medical University, Shanghai 200433, China; ^4^Institute of Nuclear Energy Safety Technology, Chinese Academy of Sciences, Hefei, Anhui 230031, China

## Abstract

With more powerful penetrability and ionizing capability, high energetic neutron radiation (HENR) often poses greater threats than photon radiation, especially on such occasions as nuclear bomb exposure, nuclear accidents, aerospace conduction, and neutron-based radiotherapy. Therefore, there emerges an urgent unmet demand in exploring highly efficient radioprotectants against HENR. In the present study, high-throughput 14.1 MeV neutrons were generated by the high-intensity D-T fusion neutron generator (HINEG) and succeeded in establishing the acute radiation syndrome (ARS) mouse model induced by HENR. A series of preclinical studies, including morphopathological assessment, flow cytometry, peripheral complete blood, and bone marrow karyocyte counting, were applied showing much more serious detriments of HENR than the photon radiation. In specific, it was indicated that surviving fraction of polydatin- (PD-) treated mice could appreciably increase to up to 100% when they were exposed to HENR. Moreover, polydatin contributed much in alleviating the HENR-induced mouse body weight loss, spleen and testis indexes decrease, and the microstructure alterations of both the spleen and the bone marrow. Furthermore, we found that the HENR-damaged hematopoiesis was greatly prevented by PD treatment in such aspects as bone marrow hemocytogenesis, splenocytes balancing, or even the peripheral blood cellularity. The additional IHC investigations revealed that PD could exert potent hematopoiesis-promoting effects against HENR via suppressing apoptosis and promoting the antioxidative enzymes such as HO-1.

## 1. Introduction

Irradiation is being applied in more and more areas such as nuclear plants, radiotherapy, and aerospace, providing us great benefits along with some adverse effects as well [1]. Especially, the neutron radiation poses severer threats than the others due to its powerful penetrability and high ionizing capability. Due to its physical trait and the key role it plays in either initiating the chain fission reactions or participating other kinds of nuclear processes, the neutron exposure exists extensively in our life, ranging from aircrew and the passengers, aerospace, nuclear reactors, particle accelerators, to radiotherapies. Thus, strategy explorations aiming at preventing human from neutron radiation injuries have been attracting a lot of attentions all the time [2].

Neutrons are classified into high linear energy transfer (LET) radiation, which means ionizing more atoms and thus producing more attacking free radicals than the low LET rays such as photons (*γ* rays or X rays) at the same situation. Indeed, numbers of studies concerning the biological effect of different types of radiation showed that the relative biological effectiveness (RBE) of neutrons was generally several times as those of low LET rays did in various models regarding with different indicators [3–5]. Furthermore, even the neutrons of different energy levels hold distinct RBEs for the same observation end point. For example, determined on human lymphocyte with either the comet assay or the chromosome aberration rates, RBE values of low-energy neutrons is higher than that of fission neutrons [6]. Thus, necessity is that the biological effect of monoenergetic neutrons should be investigated in detail separately instead of indiscriminately due to their distinct characteristics.

As a kind of noncharged particles, neutrons can penetrate materials with much depth. However, neutrons with different levels of energy have different penetrating power. The more energy confers neutrons' greater tissue-penetrating ability, which have been utilized in clinical tumor radiotherapies by neutron radiation such as boron neutron capture therapy (BNCT) regimen [2]. But as for the neutron radiation protection, it is harder to prevent the high-penetrating neutrons compared to the thermal neutrons of relatively low energy (~0.025 eV). After all, taking full advantage of the nuclear reactions prone to occur with the neutrons of different energy levels, the optimized physical shield composed of series nuclides were able to absorb most of the relatively low-energy neutrons. In contrast, high-energy neutrons can penetrate deeply and impose much threats to physical prevention, eliciting the significant demand for exploring the medical countermeasures to high energetic neutron radiation as the last line of defense [7]. Nevertheless, it is not easy to produce enough high-dose rate monoenergetic neutrons with a high level of energy to generate appropriate biological effects for medical research.

After the discovery of neutron in 1935, much progress have been made concerning the neutron biology, the main investigations of which were concentrated in RBE studies, dosimetry studies, BNCT optimizing, and some attempts to make a feel of the underlying neutron injury mechanisms [8]. However, as to the pharmacological prevention exploration, fewer studies were conducted except for several regarding cytokines and natural antioxidants [9, 10]. In the present study, we managed to establish an acute radiation sickness (ARS) mouse model irradiated by high-flux 14.1 MeV neutrons, whereby the world-leading facility named the high-intensity D-T fusion neutron generator (HINEG) [11], and made an effort to develop ideal candidate drugs for the injury.

Polydatin (PD; 3,40,5-trihydroxystibene-3-b-mono-D-glucoside), extracted from *Polygonum cuspidatum*, is a small natural compound exerting various therapeutic activities including antioxidation and anti-inflammation [12]. Previous studies have revealed that PD could also ameliorate *γ* radiation-induced injuries in multiple organs such as the lung, testis, and intestine through scavenging ROS, activating antioxidation cascades, or regulating apoptosis and many other relevant pathways [13–15]. Since neutron radiation provoked even severer toxic free radicals outbreak and apoptosis imbalance, we made an assumption that PD could still improve the ARS outcomes via its comprehensive bioactivities. Therefore, the established ARS mouse model was adopted to test whether PD administration could exert radioprotection against high-energy neutrons and to unveil the potential mechanism.

## 2. Material and Methods

### 2.1. Animals and Treatments

Male wild type 8-week old Balb/c mice were obtained from Shanghai Laboratory Animal Center of Chinese Academy of Science and maintained at 23°C to 25°C with a 12 h light/dark cycle. Before enrolling into the experiment, mice were firstly housed for a week to accommodate the new environment. All living conditions and protocols were approved by the Naval Medical University Institutional Animal Care and Use Committee in accordance with the Guide for Care and Use of Laboratory Animals published by the US NIH (publication No. 96-01).

Either PD (Sigma-Aldrich, 100 mg/kg in 0.1 ml 5% DMSO) or 0.1 ml DMSO only (5% in PBS) was delivered to the experimental mice in the corresponding groups via intraperitoneal injection one day before IR exposure and continued daily to the last. For the animal survival survey, all the mice were taken carefully and at least observed twice a day (every morning and evening) up to the 30th day post IR to make a good record of the animal state and the survival rate. As for the tissue sampling experiments, mice were anesthetized (chloral hydrate of 10% in physiological saline, intraperitoneal injection) and sacrificed to harvest different kinds of samples which was applied for the next determination on day 1, day 3, day 7, and, if still alive, day 30 post IR. Before tissue sampling, the cardiac perfusion was conducted to avoid the potential interference from blood background when employing the following experiments such as the immunohistological analysis.

### 2.2. Neutron Irradiation and Dosimetry

We adopted the high-intensity D-T fusion neutron generator (HINEG), located at The Institute of Nuclear Energy Safety Technology, Chinese Academy of Sciences, as the radiation source to provide the fast neutrons. By accelerating the deuterium ions to hit tritium targets, HINEG is designed to produce D-T fusion 14.1 MeV monoenergetic neutrons. Actually, there exited unavoidable contamination of the neutron dose with around 5% *γ* rays, whose contribution to the biological effect was yet deemed as negligible due to the higher RBE of the neutrons. Mice, which were confined in a plastic box with a certain arc ensuring each cell holding the equal distance to the neutron source, were exposed to a single dose of HENR (Figure S1).

Using the ^238^U fission chamber, the neutron yield from HINEG device has been measured as 4.00 × 10^14^ N. The absorbed dose from a beam of neutrons may be computed by considering the energy absorbed by each of the tissue elements that react with the neutrons. The type of reaction, of course, depends on the neutron energy. For fast neutrons up to about 14.1 MeV, the main mechanism of energy transfer is elastic collision. In cases of elastic scattering of fast neutrons, the scattered nuclei dissipate their energy in the immediate vicinity of the primary neutron interaction. The radiation dose absorbed locally in this way is called the first collision dose and is determined entirely by the primary neutron flux; the scattered neutron is not considered after this primary interaction. For fast neutrons, the first collision dose rate from neutrons of energy *E* is

(1) $$\begin{array}{c} {\dot{D}}_{n}\left(E\right)=\frac{\phi \left(E\right)E\sum_{i}\sigma_{i}f\;N_{i}}{1\text{J}/\text{kg}/\text{Gy}}, \end{array}$$

where  *ϕ*(*E*) is the flux of neutrons whose energy is *E*, in neutrons per cm^2^ per second, *σ*_*i*_ is the scattering across section of the *i*th element for neutrons of energy *E*, in barns ×10^−24^ cm^2^, *f* is the mean fractional energy transferred from neutron to scattered atom during collision with the neutron, and *N*_*i*_ is the number of atoms per kilogram of the *i*th element.

According to the neutron influence of the sample, the calculated absorbed doses of each group were 0.64 Gy, 0.95 Gy, 1.54 Gy, and 2.91 Gy, respectively (see more details in the Table S2).

(2) $$\begin{array}{c} \text{Total uncertainty}=\sqrt{\text{distance uncertaint}{\text{y}}^{2}+\text{distance uncertaint}{\text{y}}^{2}+\text{yield uncertaint}{\text{y}}^{2}}. \end{array}$$

### 2.3. Biometric Parameters Determination

Body weight of each mouse was measured every 3 days until they were sacrificed. The body weight curves were generated based on the average and standard error of mean (SEM) of each group. To calculate the spleen and testis indexes, both of the above were excised and weighted after the mice were killed. The following formula was adopted to get the final result:

(3) $$\begin{array}{c} \text{Organ index}=\frac{\text{organ weight}\left(\text{g}\right)}{\text{body weight }\left(\text{g}\right)}\times 1000\text{‰}. \end{array}$$

### 2.4. Peripheral Complete Blood Count Analysis

Immediately after the mice were anesthetized by an anesthesia apparatus (Norvap, U.K.) with isoflurane, blood samples (0.7 ml) were obtained from the angular vein and collected into the ethylenediaminetetraacetic acid-coated anticoagulant tubes for the following analysis via an automatic blood cell analyzer (Mindray, Shenzhen, China) according to the manufacture's instruction. Then, a comprehensive result involving WBCs, RBCs, PLTs, and their subsets was outputted and investigated.

### 2.5. Marrow Karyocyte Counting

Left femur of each sacrificed mouse was holed on both sides and washed repeatedly with 1 ml PBS for 3 times until the femur turned white. Then, the cell suspension was filtered through 100 *μ*m cell strainer (BD FALCON, New Jersey USA) and centrifuged at 1500 rpm for 5 min. With the supernatant discarded, the pellet was lysed with 1 ml Red Blood Cell Lysis Buffer for 10 minutes to remove the erythrocytes, leaving the bone marrow nucleated cells which were washed and eventually resuspended with 1 ml PBS. Flow cytometry was then performed to enumerate the total nucleated cells of each femur within 1 min at the sample flow rate of 10 *μ*l/min.

### 2.6. Splenocyte Apoptosis

The mouse spleen was isolated and ground with a metal bar on a 200-mesh metal net, and then, the single cell suspension was made. After being washed with 1 ml PBS twice, the cells were firstly incubated with FITC for 30 minutes and then stained with PI dye according to the manufacturer's operating manual (TransGen Biotech, Beijing, China). Flow cytometry was adopted to measure the apoptotic performance of each group. At least 10,000 gated events were collected and analyzed for each sample.

### 2.7. Histopathology and Morphometry

Spleens and femurs were removed and fixed with 4% paraformaldehyde for at least 24 hours. Next, the samples were embedded in paraffin and cut into thin sections (4 *μ*m thick) for the next staining analysis. We applied the hematoxylin and eosin staining (H&E) to conduct the regular histopathological microstructure discrimination. To determine the specific molecular alteration, we performed the immunohistochemistry analysis (IHC) using the according antibodies: anti-Heme oxygenase-1 (HO-1), 1 : 1000; anti-Keltch-like ECH-associated protein 1 (KEAP 1), 1 : 1500; and anti-sirtuin 1 (SIRT 1), 1 : 500. The H&E slides were investigated under a microscope (Nikon, T1-SAM, Japan) adapted with a CCD camera (Nikon, DS-Ri2, Japan) while the IHC slides were scanned using an automatic digital slide scanner (Pannoramic MIDI, 3D HISTECH, Hungary). A quantitative analysis of the IHC pictures was performed using the IHC profiler plugin in the ImageJ software [16, 17].

### 2.8. Western Blot Assay

At the indicated time points, animals were sacrificed and the lung tissue was isolated and rapidly frozen in liquid nitrogen; then, they were stored at -80°C. The protein was extracted by using M-PER mammalian protein extraction reagent (Thermo Fisher Scientific) according to the manufacturer's instruction. After blocking for 1 hour at room temperature, the membranes were probed overnight at 4°C with the primary antibodies such as Bcl2 (Cell Signaling Technology, 1 : 1000) and Actin (Proteintech, 1 : 1000), and then the secondary antibody (Cell Signaling Technology, 1 : 5000).

### 2.9. Statistical Analysis

All data were presented as mean ± SEM, and the statistical analysis was carried out using the SPSS 22.0 software (SPSS Inc., Chicago, USA). The GraphPad Prism 6 Software (GraphPad Software Inc., California, USA) was utilized to make the graphs. As for the survival rate comparison, the Kaplan-Meier method and the following log-rank test were performed to determine the significance. Besides, statistical significances between two groups were determined by Student's *t*-test. Differences were considered to be statistically significant when the *p* value was less than 0.05.

## 3. Results

### 3.1. Animal Survival

**Figure 1:**
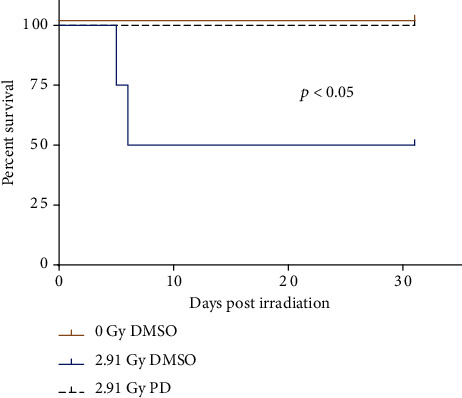
Survival curves of different treatment groups. *n* = 10; *p* < 0.05: 2.91 Gy DMSO vs. 2.91 Gy.

Twenty-four Balb/c mice were randomly assigned to different groups (8 mice for each) including 0 Gy DMSO group, 2.91 Gy DMSO group, and 2.91 Gy PD group. The animal performance was observed closely, and the survival curve was obtained. Although the upmost neutron radiation dose was adopted for this experiment, for which the animal tolerance to the circumstances was prudently considered, merely about half of the whole mice died until the end of one month after IR exposure (Figure 1). In sharp contrast, none of the 2.91 Gy PD group mice died during the observation term just as the nonradiation group did.

### 3.2. Biometric Parameters

**Figure 2:**
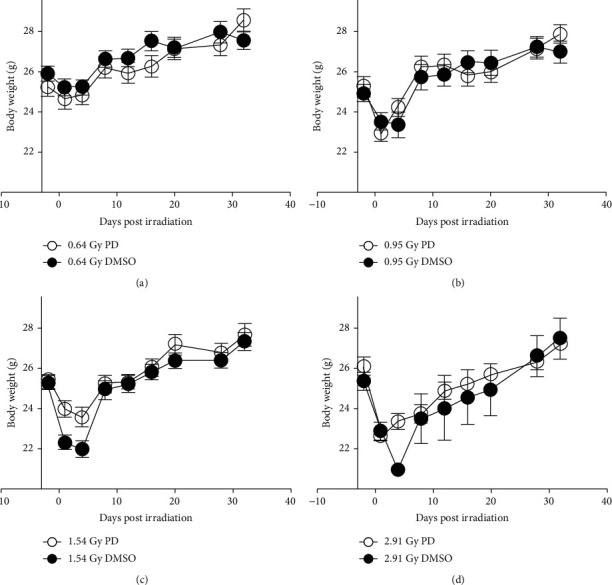
Acute mouse body weight loss due to 1.54/2.91 Gy HENR exposure was prevented greatly by PD. Body weight of every mouse was consistently measured ever few days till D32 post IR, and the alteration curves of each group was plotted here (a–d). *n* = 8, ∗*p* < 0.05, ∗∗*p* < 0.01, ∗∗∗*p* < 0.001.

Biometric parameters of the experimental mice were measured 1 day, 3 days, 7 days, and 32 days after neutron irradiation. Mice, which were sacrificed on D32, received 0.64 Gy, 0.95 Gy, 1.54 Gy, and 0 Gy radiation dose while the other animals were only exposed to 2.91 Gy radiation (Table 1). Considering the IR caused potential deaths of the mice, we assigned another 10 mice into the 2.91 Gy IR DMSO group. The animal body weight from all D32 groups of Table 1 combined with the ones in the above survival analysis was all tracked, and the data were analysis integrated. Finally, at least 8 mice for each group were enrolled into the body weight statistics. As depicted in the curves, no matter which of the IR dose was chosen, each of the experimental groups exhibited a first decrease and then increase tendency of body weight alteration (Figures 2(a)–2(d)). Particularly, when the doses rose up to 1.54 Gy or 2.91 Gy, a statistic significance with or without PD administration showed several days after IR (Figure 2(c): *p* = 0.00782522, *p* = 0.0253507; Figure 2(d): *p* = 0.00115645).

**Figure 3:**
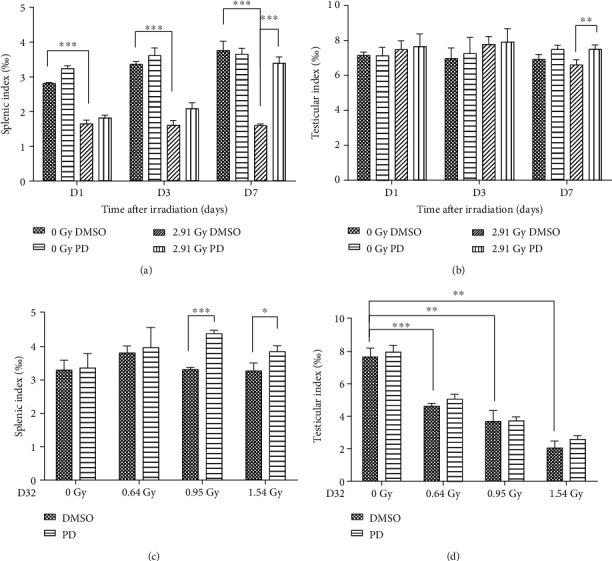
PD attenuated both the splenic and testicular index of the irradiated mice. (a, b) The splenic and testicular indexes were calculated at different time points (D1, D3, and D7) post 2.91 Gy neutron radiation. (c, d) On the 32th day after exposing to a variety of radiation doses (0.64 Gy, 0.95 Gy, 1.54 Gy, and 0 Gy as sham), the organ indexes of the above were analyzed again. ∗*p* < 0.05, ∗∗*p* < 0.01, ∗∗∗*p* < 0.001.

The irradiated groups including the 0.64 Gy, 0.95 Gy, and 1.54 Gy dose groups for D32 and the 2.91 Gy groups for D1, D3, and D7 were subjected to the organ index determination (Figure 3). In terms of the spleen, the organ index dropped sharply a relatively short period after IR (Figure 3(a), D1: *p* = 0.00023741, D3: *p* = 0.000236877, D7: *p* = 0.000235673). As compared to the IR + DMSO groups, this damaged parameter was turned by PD to be better and better as time went by, with a significance showing on D7 (*p* = 0.000113204). On the 32th day post IR, the damaged splenic index caused by IR seemed to be restored to the normal level. Moreover, PD administration elevated this index markedly in the 0.95 Gy and 1.54 Gy groups comparing with their corresponding IR + DMSO treatment group (0.95 Gy, *p* = 0.000115282; 1.54 Gy, *p* = 0.0138295). As for the testis, the index was decreased by IR on D7, which was rectified by PD greatly (*p* = 0.00348368), while no significances were observed among the other comparisons (Figure 3(b)). However, a clear dose-dependent reduction showed up among the IR + DMSO groups 32 days post IR, with a slight elevation observed in the PD treated groups (Figure 3(d)).

### 3.3. Bone Marrow Nucleated Cells

Mice which were injected with 5% DMSO only or containing PD were exposed to 2.91 Gy neutron radiation or sham dose. Left femur of mice was isolated, and their bone marrow was flushed for BMNC assessment. Under IR exposure, the IR DMSO groups lost much of the BMNC compared with the DMSO groups at the corresponding time points (D1: *p* = 0.0137359; D3: *p* = 0.000480536; D7: *p* = 0.00008394891). Thanks to the PD administration, the IR-compromised BMNC of each IR DMSO group was greatly restored on D1 (*p* = 0.026204) and D3 (*p* = 0.000321216). Additionally, it seemed that the BMNC of nonradiated groups, including both the DMSO group and the PD group, began to decline on D7 as relative to the previous time points, which should be attributed to the hematopoietic toxicity of the solvent DMSO.

### 3.4. Peripheral Hematological Studies

To determine the ability of PD to ameliorate radiation-induced defects in hematopoiesis, the mouse peripheral complete blood count analysis (CBC) was carried out on D1, D3, and D7 post 2.91 Gy neutron radiation.

**Figure 4:**
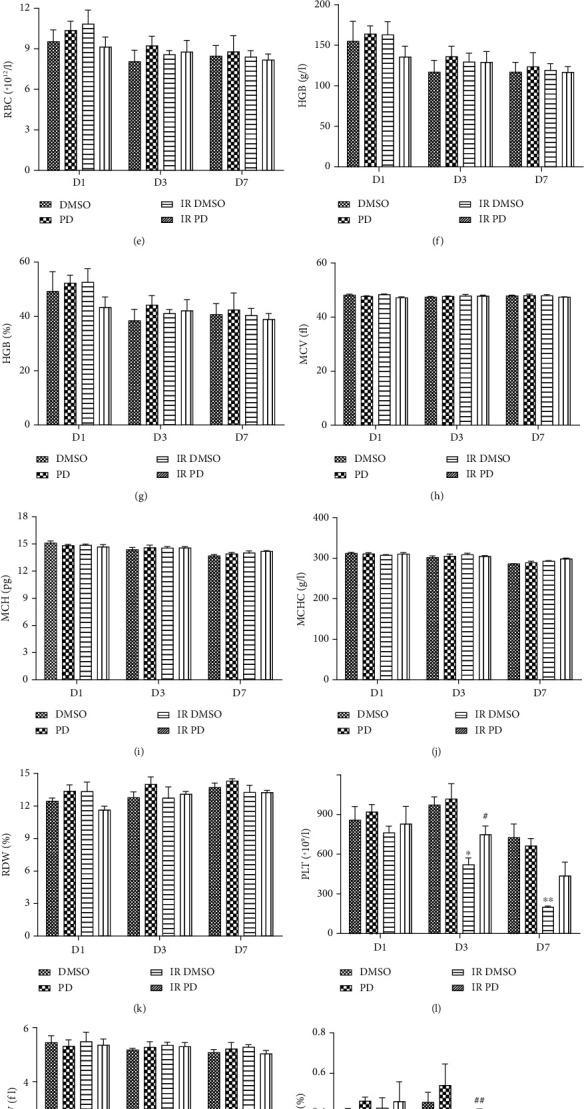
(a–d) Circulating WBC and its subsets of mice with/without PD administration in combination with IR or not. (a–d) WBC, Lymph, Mon, and Gran counts, respectively. Data are mean ± SEM, *n* = 8. ∗*p* < 0.05, ∗∗*p* < 0.01, and ∗∗∗*p* < 0.001 vs. the DMSO group; ^#^*p* < 0.05 and ^##^*p* < 0.01 vs. the IR DMSO group. (e–k) Circulating RBC and the related parameters are shown here. (e–k) RBC, HGB concentration, HGB percentage, MCV, MCH, MCHC, and RDW, in sequence. Data are mean ± SEM, *n* = 8. (l–n) The alterations of PLT and the relevant indicators in peripheral vessels of mice. (l–o) PLT, MPV, PCT percent, and PDW, sequentially. Data are mean ± SEM, *n* = 8. ∗*p* < 0.05, ∗∗*p* < 0.01, and ∗∗∗*p* < 0.001 vs. the DMSO group; ^##^*p* < 0.01 vs. the IR DMSO group.

As seen in Figures 4(a)–4(d), the white blood cells (WBC), lymphocytes (Lymph), Monocytes (Mon), and the Granulocyte (Gran) shared a similar alteration style to IR injury, declining at all the three time points with the lowest count on D3. As for the WBC (Figure 4(a)), the differences were all considerable all through the time course (*p* = 0.00343867 for D1, *p* = 0.00270821 for D3, and *p* = 0.00321815 for D7), with an evident amelioration effect of PD treatment showing up simultaneously (*p* = 0.00246184 for D3 and *p* = 0.0129618 for D7). As the major component of WBC, lymph count also descended quickly and sharply after IR (*p* = 0.0103933 for D1, *p* = 0.0111376 for D3, and *p* = 0.0050193 for D7, compared to DMSO groups, respectively). PD rescued this defect as well, with a significance observed on D3 (*p* = 0.00168689 for D3, in comparison with the IR DMSO group). Moreover, PD seemed to improve the IR-suppressed Mon level on D1 and D7. However, the Mon number was too few to be detected on D3 for both of the IR groups.

Figures 4(e)–4(k) depict the results of the red blood cells (RBC), the hemoglobin (HGB), the mean corpuscular volume (MCV), the mean corpuscular hemoglobin (MCH), the mean corpuscular hemoglobin concentration (MCHC), and the red blood cell distribution width (RDW) in mouse peripheral blood. Unlike the WBCs, no marked changes were found regarding this panel.

Three days after IR, the PLT value reduced significantly (*p* = 0.0115232) and continued to fall much lower level on D7 (*p* = 0.00174034). However, treatment with PD increased the PLT count in irradiated mice compared to irradiated mice (*p* = 0.0479962 for D3). Similar to this is the PCT performance, with a significant difference between IR DMSO and IR PD groups on D3 (*p* = 0.00671847). Though the MPV data told us the platelets of different groups should have similar individual volume (Figure 4(m)), the PDW level was apparently elevated by IR over time (D7, *p* = 0.000368411) which was attenuated again by PD treatment (D7, *p* = 0.00154739; Figure 4(o)).

### 3.5. Apoptosis

**Figure 5:**
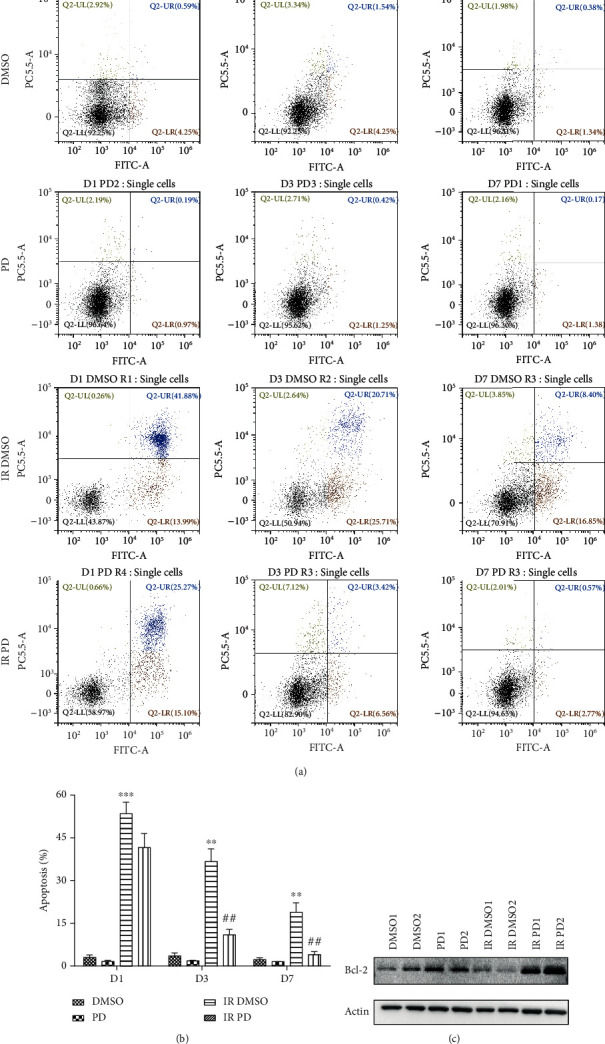
Mouse splenic and lung apoptosis changes after IR/sham combined with/without PD administration. (a) Representative dot plots chosen randomly from each treatment group. The *X* axis indicates FITC fluorescence intensity, and the *Y* axis represents PI fluorescence intensity (as the data were generated via the Beckman Coulter flow cytometry (CytoFLEX) and analyzed by the CytoExpert software, of which the channel of PC5.5 covers the wavelength of PI dye, and the channel name is PC5.5). The events of each quadrants were discriminated with different colors with the corresponding percentage showing in each corner. (b) The calculated statistical data from three independent identical experiments. Data are mean ± SEM, *n* = 8. ∗∗*p* < 0.01 and ∗∗∗*p* < 0.001 vs. the DMSO group; ^##^*p* < 0.01 vs. the IR DMSO group. (c) Immunodetection of mouse lung tissue from different treatment groups. Two samples were randomly selected from each group and underwent the WB analysis. For each well, 15 *μ*g protein were loaded here.

The spleen cells' apoptosis rate was evaluated by flow cytometry using an Annexin V/PI staining kit. The representative pictures of dot plot in Figure 5(a) were divided into four quadrants including the early apoptosis quadrant (LR) and the late apoptosis quadrant (UR), the sum of which indicated the total apoptosis rate. From the dot plots, we can achieve a direct assessment that at whichever time points, the combined dots of LR and UR quadrants turned to be much more as the individuals were irradiated, and this trend was partly prevented by the PD treatment (Figure 5(a)). Additionally, the summarized data of three independent repetitive experiments provides us with more specific statistics (Figure 5(b)). Under irradiation exposure, the percentage of apoptosis surged greatly in comparison with the corresponding DMSO groups (D1, *p* = 0.000135389; D3, *p* = 0.0015616; and D7, *p* = 0.0081346), while PD administration attenuated this disorder a lot thereafter (D3, *p* = 0.00542718; D7, *p* = 0.00459456). Moreover, it was suggested that the IR boosted splenic cell apoptosis rate reached the highest peak on D1, then descended gradually over time, showing the characteristic spleen cell apoptosis response style to neutron irradiation stress (Figure 5(b)).

To explore the apoptosis alteration more comprehensively, we applied the WB method to the mouse lung tissue to evaluate the antiapoptosis molecular Bcl-2 level. As depicted in Figure 5(c), IR deregulated the bcl-2 expression obviously. In contrast, PD administration reverted this change greatly, enhancing this key antiapoptosis regulator as much.

### 3.6. Histological Examination

**Figure 6:**
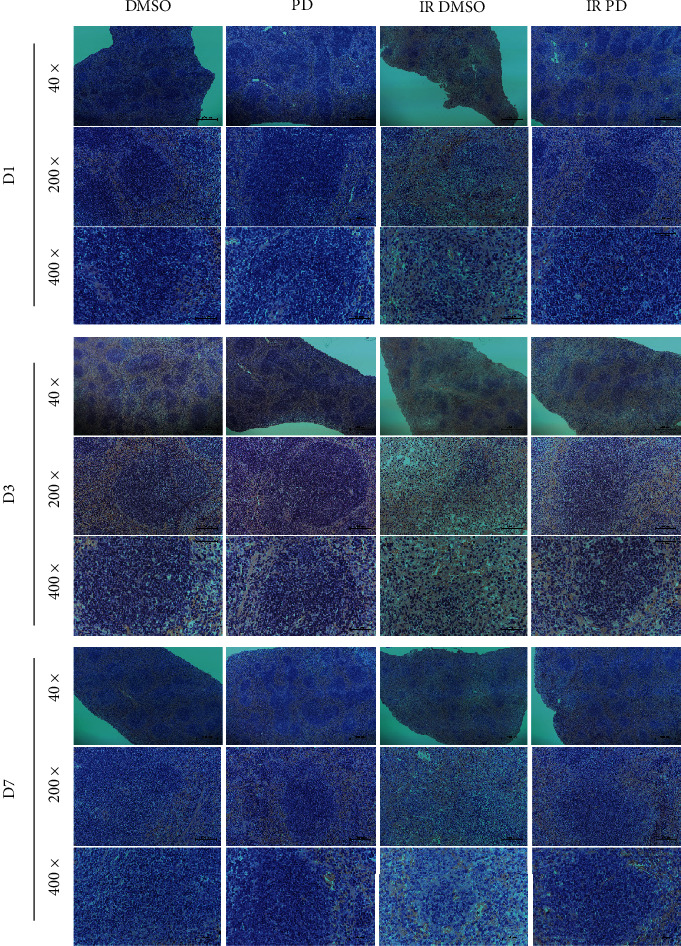
Histology analysis of mouse splenic tissue from different groups. The spleens from 3 mice every group were fixed in 4% formaldehyde and embedded in paraffin. Sections were stained with Hematoxylin–Eosin, and histological examination were applied.

**Figure 7:**
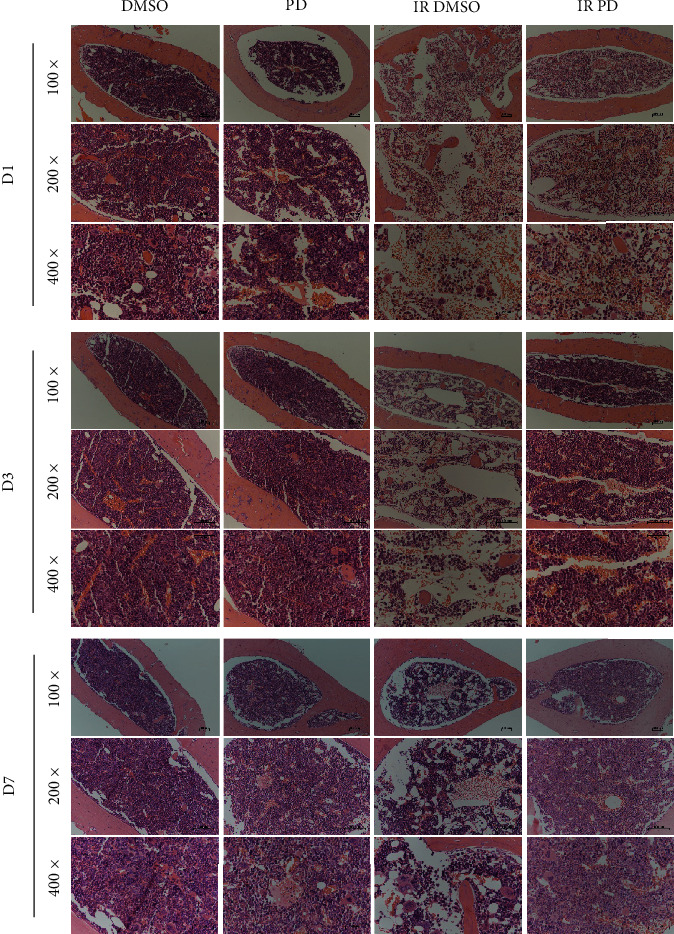
Histology analysis of mouse bone marrow from different groups. Femurs from at least 4 mice every group were isolated and fixed in 4% formaldehyde and embedded in paraffin. Sections were stained with Hematoxylin–Eosin, and histological examination was applied.

The spleen and bone marrow from all the groups of mice were dissected and histopathologically examined at different time points along with the study. We conducted the microscopic examination at various magnification (Figures 6 and 7). As for the spleen, with the low-power objective, we found that the density of the white pulps of the spleen turned much lower after the IR exposure all through the experiment course. Furthermore, the white pulps lost the normal microstructure compared to the DMSO/PD groups which held much clearer bolder called the marginal zone. The high-power objective presented pictures with much more details showing that the overall cellular density of the radiated spleen was reduced greatly and most of the white pulps atrophied and became disorganized. Taking all the above observation outcomes into consideration to compare the IR groups on different days, we can see that the spleen got mostly damaged on D3 and recovered a little on D7. However, the histological investigation revealed that all the deleterious effects of neutron radiation on the spleen microstructure were evidently relieved under the PD administration. With respect to the bone marrow, similar histological alterations were observed as that of the spleen (Figure 7). Neutrons seriously destroyed the microstructure of the bone marrow, causing vacuoles and disorganization, and greatly reduced the bone marrow cells, especially the series of the hematopoietic progenitor cells. In contrast, though the PD treated mice were also exposed to the same neutron radiation dose, their bone marrow specimen nearly looked the same as the sham-radiated groups, indicating that PD exerted strong hematopoietic process enhancement which helped the organism undergo the IR-induced crisis.

### 3.7. Immunohistochemistry Analysis

**Figure 8:**
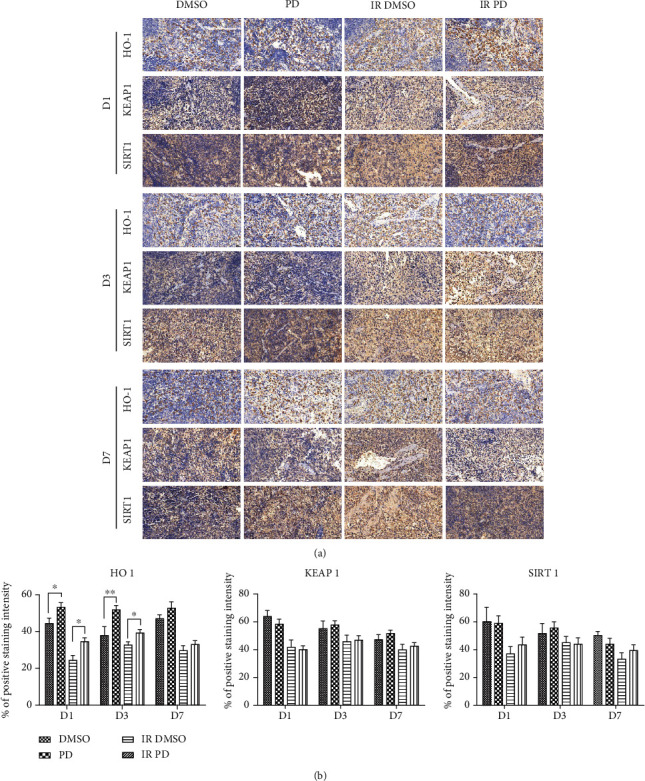
Immunohistochemistry analysis of HO-1, KEAP 1, and SIRT 1 using mouse spleen from different groups. The spleens from at least 4 mice each group were isolated and fixed in 4% formaldehyde and embedded in paraffin. Sections were stained with DAB and incubated with the corresponding antibodies. A whole slide scanning method was utilized to obtain the digital pictures which were then applied to calculate the IHC staining intensity score via the IHC profiler plugin in the ImageJ software. (a) Representative IHC images of each group; (b) quantified percentage score of IHC pictures from at least eight high-power fields (×400) every group was subjected to statistical analysis using a two-tailed unpaired *t*-test. The experiments were performed in triplicates, and data are presented here as mean ± SEM. ∗*p* < 0.05, ∗∗*p* < 0.01.

To explore the potential mechanism of the radioprotective effects of PD against neutron radiation detriments, we applied the IHC analysis for the detection of HO-1, KEAP 1, and SIRT 1. However, the splenic cellularity was so high that manual investigation and scoring of the IHC pictures would be more error prone due to the subjectivity of different pathologists. Hence, we conducted this part by the ImageJ software equipped with IHC profiler plugin. Although the representative images did not present a sharp visual contrast of positive staining occupation among different groups, significant differences were still observed after statistical calculation (Figure 8). For example, HO-1 expression was marked elevated by PD administration on D1 and D3 both pre- (*p* = 0.0453801 for D1 and *p* = 0.00783043 for D7) and post IR (*p* = 0.00432644 for D1 and *p* = 0.0142722 for D7). As for both KEAP 1 and SIRT 1, there were no obvious changes between groups according to the statistical analysis. Though not significant, a slight increase of SIRT 1 expression after PD administration was still noticed here.

## 4. Discussion

**Figure 9:**
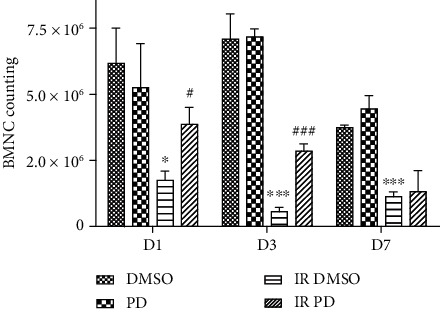
PD enhanced the IR-damaged BMNC. The IR dose with 2.91 Gy. DMSO with or without PD was administrated once a day until sacrifice. Data are mean ± SEM, *n* = 8. ∗*p* < 0.05 and ∗∗∗*p* < 0.001 vs. the DMSO group; ^#^*p* < 0.05 and ^###^*p* < 0.001 vs. the IR DMSO group.

In this study, a neutron radiation-induced ARS mouse model was adopted to determine whether the potent natural antioxidant PD was able to prevent the severe injuries. The convincing data here positively favored this assumption, showing that the PD administration improved the survival rate after IR and ameliorated the IR-induced body weight loss and spleen and testis shrinking (Figures 1–3). In advance, obvious evidence indicated that PD could powerfully enhance both intra- and extramedullary hematopoiesis recovery against neutron detriments and present much better performance in the peripheral whole blood counting test (Figures 4 and 9), which was further ascertained by histological analysis of both the spleen and bone marrow (Figures 6 and 7). Additionally, it was suggested that the regulation of apoptosis and antioxidant signal pathway may play a vital role in PD's radioprotection in hematopoiesis (Figures 5 and 8).

Since the experimental discovery of neutron in 1932, its biological effects have attracted great interest, and there have been a mountain of related researches regarding neutron biology, among which the monoenergetic neutrons were paid more and more attention as it is more feasible and rational to explore the mechanism of the bioresponse to neutrons using single levels of energy instead of the sophisticated mixture [2]. Due to the limitation of the neutron flux of the previous irradiators, it was hard to make the high monoenergetic neutron-induced ARS animal model because of the conflict between the restrained duration for animal exposure and the insufficient dose rate. However, we adopted the HINEG system, which can present the high flux of 14.1 MeV neutrons as to generate the mouse ARS model utilized here, to conquer this obstacle. Compared with our previous studies about ionizing photons protection, similar phenomena such as survival rate decline, body weight loss, critical organ shrinking, and hematopoietic tissue cell loss were also obvious in the neutron ARS model here [18, 19]. The detriments by neutrons were much severer than that of photons, characterized by relatively low dose causing the similar outcomes and the advanced upcoming of injury peak, all of which promoted the insight of neutron biology at this condition.

In the current study, PD restored the survival rate from less than 50% up to 100%, showing its powerful protective capacity against neutrons (Figure 1). Indeed, it would be more convincing if the neutron exposure could sacrifice most of the subjects. Therefore, we will increase the total dose by enhancing the neutron flux after the facility is upgraded in the near future. Body weight decreased obviously on the first few days after IR on a dose-dependent style, with the lowest average bodyweight value declining as the doses climbing (Figure 2). However, PD administration lessened the primary body weight loss significantly, reflecting its efficacy to helping getting through the critical early phase of ARS (Figure 2).

As is well known, injuries to the hematopoietic system is one of the major contributing factors for the ARS progression, resulting in the hematopoietic subsyndrome due to its profound detriments to the actively proliferating progenitor cells [20, 21]. For a long time, the main countermeasure against IR was to seek protective strategies to preserve damaged hematopoietic precursor cells or restore their function [22]. Recently, the powerful effect of resveratrol to restore the pancytopenia induced by ionizing photons was observed [23]. As a precursor of resveratrol, PD exerts many similar biomedical properties such as antiplatelet aggregation, antioxidative action, cardioprotective activity, anti-inflammatory, and immune-regulating functions [24]. In the present study, PD elevated the IR-injured BMNCs (Figure 9) and mitigated the cellularity and microstructure of the bone marrow (Figure 7) which is the major source of blood cells and, as expected, alleviated the peripheral blood cell decline involving lymphocytes, granulocytes, WBCs, and PLTs (Figure 4). Additionally, identical tendency was found in the spleen specimen examination (Figure 6). Besides, all of the above indicators followed the same trend over time with the lowest value emerging on D3, except that PLT value entered into its bottom on D7 (Figure 4). This is probably due to the longer longevity and less radiosensitivity of PLTs compared to lymphocytes, causing the relatively delayed decline of PLTs in the peripheral blood.

Apoptosis surge is one the most important functioning manners of the IR cellular toxicity, so firstly, we conducted apoptosis detection for nucleated splenic cells to explore the potential mechanism. After IR, the apoptosis rate increased immediately and reached the top value on D1, followed by slightly continuous decreases on D3 and D7 (Figure 5). Collectively, the apoptotic nucleated splenic cells soared to the culmination immediately after IR and apparently responded quicker than the hematologic manifest. After all, it took some time from the onset of apoptosis to cell death, for which there existed the delay of the hematologic manifest. Nevertheless, PD alleviated the apoptosis greatly in spite of the time points with statistical differences on D3 and D7. What is more, WB data favored PD's antiapoptotic role in its radioprotection as well (Figure 5(c)). However, more extensive investigations such as detection of other apoptosis-relevant effectors should be considered to verify the antiapoptosis role of PD against HENR in the next study.

Previous studies indicated that PD could prevent neurons and hepatocytes by increasing the expression and activity of SIRT 1, which is very important for the function of hematopoietic stem cells [23, 25, 26]. As PD showed great performance in alleviating hematopoietic function injuries induced by HENR, we firstly hypothesized that SIRT 1 activation might improve hematopoiesis. However, from the IHC results, we found that PD merely elevated SIRT 1 positive staining slightly. Similarly, no significant alterations were observed in the KEAP 1 IHC analysis, which is an important inhibitor of the antioxidant gene Nrf 2. Taking the severe cell deaths caused by HENR into consideration, the IHC data reflected a comprehensive effect of the whole spleen cells, in which the former may play an even more important role resulting uniformly decline of positive staining after HENR regardless of protein targets. Even for the strong antioxidant enzyme, HO-1 were found decreased by HENR, but it was clearly raised by PD both pre- and post-HERN, suggesting a cytoprotective role of PD against various oxidative stress and inflammatory responses [27]. However, considering the confounding factors such as HENR-induced cell deaths mentioned above and the cellular heterogeneity of the in vivo studies, the more specific methods aiming directly at specific sorts of cells would be adopted in the future mechanism-exploration research.

In conclusion, the present study demonstrated that PD played a protective role against neutron IR injuries in mice by accelerating hematopoiesis, suppressing apoptosis of nucleated blood cells, and regulating the antioxidative function such as the HO-1 pathway. Our findings may throw light on some characterized bioeffects induced by the high flux of 14.1 MeV neutrons and propose that PD can powerfully mitigate these damages, indicating its potential role of new strategy for the prevention of high-energy neutron IR detriments.

## Figures and Tables

**Table 1:** List of the experimental animal groups with the according treatments.

Time post IR	Treatment	Explanation
D1	DMSO	Nonirradiated mice with DMSO administration for 1 day
	PD	Nonirradiated mice with PD administration for 1 day
	IR + DMSO	Irradiated (2.91 Gy) mice with DMSO administration for 1 day
	IR + PD	Irradiated (2.91 Gy) mice with PD administration for 1 day
D3	DMSO	Nonirradiated mice with DMSO administration for 3 days
	PD	Nonirradiated mice with PD administration for 3 days
	IR + DMSO	Irradiated (2.91 Gy) mice with DMSO administration for 3 days
	IR + PD	Irradiated (2.91 Gy) mice with PD administration for 3 days
D7	DMSO	Nonirradiated mice with DMSO administration for 7 days
	PD	Nonirradiated mice with PD administration for 7 days
	IR + DMSO	Irradiated (2.91 Gy) mice with DMSO administration for 7 days
	IR + PD	Irradiated (2.91 Gy) mice with PD administration for 7 days
D32	DMSO	Nonirradiated mice with DMSO administration for 32 days
	PD	Nonirradiated mice with PD administration for 32 days
	IR + DMSO	Irradiated (0.64 Gy, 0.95 Gy, 1.54 Gy, and 2.91 Gy as indicated, respectively) mice with DMSO administration for 32 days
	IR + PD	Irradiated (0.64 Gy, 0.95 Gy, 1.54 Gy, and 2.91 Gy as indicated, respectively) mice with PD administration for 32 days

## Data Availability

All the data used to support the findings of this study were supplied by the Naval Medical University and Chinese Academy of Sciences in China under license and so cannot be made freely available. Requests for access to these data should be made to Dr. Guo via mailing to jiamingguonmu@smmu.edu.cn.

## Supplementary Materials

Supplementary Materials Figure S1: pictures of confined mice which were ready to receive HENR. Table S2: table of calculated dose values based on the straight-line distance to the radiation source.
