# Supplementary material for: Polydatin Attenuates 14.1 MeV Neutron-Induced Injuries via Regulating the Apoptosis and Antioxidative Pathways and Improving the Hematopoiesis of Mice
Source: Oxid Med Cell Longev. 2020 Aug 31;2020:8905860. doi: 10.1155/2020/8905860 (PMC7479486; doi:10.1155/2020/8905860)
Supplement: Supplementary Materials — Figure S1: pictures of confined mice which were ready to receive HENR. Table S2: table of calculated dose values based on the straight-line distance to the radiation source. [file 8905860.f1.pdf]

Supplement Material:

Supplement material 1: pictures of confined mice which were ready to receive HENR.

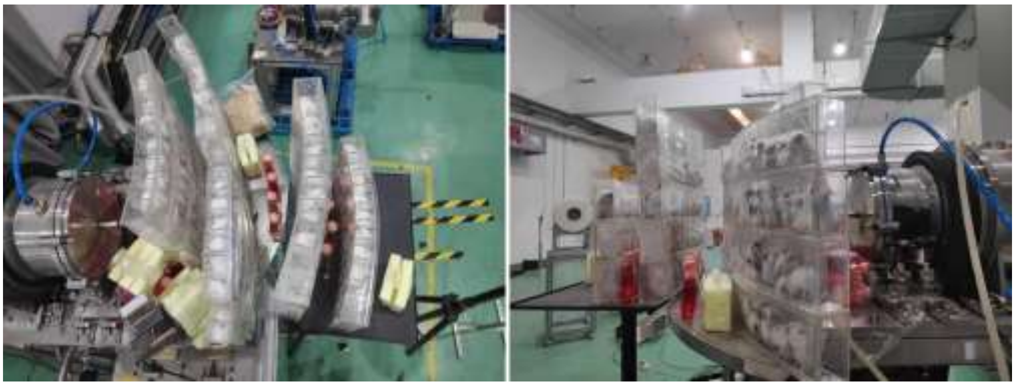

Supplement material 2: Table of calculated dose values based on the straight-line distance to the radiation source.

Sample	Distance(cm)	Total Uncertainty	Neutron Influence (10^{14} n/m^2)	Absorbed Dose (Gy)
Balb/c mice	26.6±3.0	17.05 %	4.5±0.77	2.91±0.50
	36.6±2.2	10.43 %	2.38±0.25	1.54±0.17
	46.6±1.8	8.13 %	1.47±0.12	0.95±0.08
	56.6±1.5	7.09 %	0.99±0.07	0.64±0.05
